# Quality of Life During Chemotherapy for Breast Cancer in a West African Population in Dakar, Senegal: A Prospective Study

**DOI:** 10.1200/JGO.19.00106

**Published:** 2019-07-19

**Authors:** Domitille Dano, Clémence Hénon, Ousseynou Sarr, Kanta Ka, Mouhamadou Ba, Awa Badiane, Ibrahima Thiam, Papa Diene, Mamadou Diop, Ahmadou Dem, Patricia Marino, Julien Mancini, Pierre Annede, Anthony Gonçalves, Doudou Diouf, Audrey Monneur

**Affiliations:** ^1^ Institut Paoli-Calmettes, Marseille, France; ^2^ Institut Gustave Roussy, Villejuif, France; ^3^ Aristide Le Dantec Hospital, Dakar, Senegal; ^4^ Aix Marseille University, Marseille, France; ^5^ Timone University Hospital, Marseille, France

## Abstract

**PURPOSE:**

The prevalence of breast cancer is increasing in low- to middle-income countries such as Senegal. Our prospective study assessed the quality of life (QoL) of patients with breast cancer undergoing chemotherapy in Senegal.

**PATIENTS AND METHODS:**

Our study included women with breast cancer undergoing chemotherapy as initial treatment at the Center Aristide Le Dantec University Hospital in Dakar. Clinical, sociodemographic, and QoL data were collected and analyzed at three different times: baseline, 3 months, and 6 months after the start of systemic therapy. Health-related QoL was assessed using a Functional Assessment of Cancer Therapies-Breast (FACT-B) questionnaire after translation into the Wolof language. Linear mixed-effects models were performed to assess the changes in QoL scores.

**RESULTS:**

Between July 2017 and February 2018, 120 patients were included in the study. Their median age was 45 years. Most patients (n = 105; 92%) had locally advanced disease (T3 to T4 stage) and lymph node involvement (n = 103; 88%), and half had metastatic disease. The FACT-B total scores significantly improved over time (β = 1.58; 95% CI, 0.50 to 2.67; *P* < .01). Nausea and vomiting were significantly associated with a decrease in FACT-B total scores (β = −16.89, 95% CI, −29.58 to −4.24, *P* = .012; and β = −13.44, 95% CI, −25.15 to −1.72, *P* = .028, respectively).

**CONCLUSION:**

Our study confirmed the feasibility of standardized QoL assessment in Senegalese patients with breast cancer. Our results indicated a potential improvement of QoL over the course of chemotherapy. Optimizing nausea and vomiting prevention may improve QoL.

## INTRODUCTION

The prevalence of breast cancer (BC) is increasing in low- to middle-income countries, such as those in West Africa. According to the GLOBOCAN 2018 study,^1^ the incidence of BC reached 1,758 cases per year in Senegal (compared with 869 in 2012). However, these statistics seem to be largely underestimated for several reasons, including poor reporting processes, lack of cancer registries, lack of diagnostic facilities, and low accessibility to screening and oncology care in rural areas.

**Context**

**Key Objective**
How does quality of life (Qol) evolve in patients with breast cancer during chemotherapy in low- to middle-income countries such as Senegal?
**Knowledge Generated**
After translating the Functional Assessment of Cancer Therapies-Breast QoL questionnaire into Wolof, the main language spoken in Senegal, we assessed the evolution of QoL during chemotherapy and observed improvements in QoL over time. However, the onset of chemotherapy-related adverse events, particularly nausea and vomiting, were significantly associated with a decrease in QoL.
**Relevance**
Our results highlight the potential improvement of QoL over the course of chemotherapy and the need for optimization of supportive care, including the prophylaxis of nausea, to further improve QoL.

Few medical care units or centers devoted to cancer exist in Senegal. Most patients with BC are treated at the referral center Aristide Le Dantec University Hospital Center (UHC) in Dakar, which sees 350 new patients per year. The remaining patients are referred to either Principal Hospital, the second largest UHC in Dakar, or to small public hospitals and private centers. Of note, patients are wholly responsible for the costs of medical care, including in the case of cancer.

Several potential issues in BC management may affect this population’s quality of life (QoL), including financial difficulties, limited access to treatment, and late diagnosis, among others. Importantly, the perception of disease and social constraints may differ between countries, strongly influencing QoL,^2^ cancer treatments, and types of supportive care required.

Developing an objective QoL assessment tool is crucial in adapting cancer care to a population’s specific needs. Several QoL questionnaires are suitable for patients with cancer, the most widespread tools being Functional Assessment of Cancer Therapies^3^ and European Organisation for Research and Treatment of Cancer Quality of Life Questionnaire C30.^4^ However, any QoL assessment tool must first be translated into the studied population’s native language, followed by cross-cultural validation.^5^ In Senegal, although French is the official language, Wolof is estimated to be the most used language, spoken by nearly 80% of the population.

The purpose of this study was to assess QoL of patients with BC while undergoing chemotherapy in Senegal, via Functional Assessment of Cancer Therapies-Breast (FACT-B) questionnaires translated into Wolof.

## PATIENTS AND METHODS

This prospective study included patients diagnosed with BC and treated at the Aristide Le Dantec UHC in Dakar, Senegal. Inclusion criteria included histologically or cytologically confirmed BC and indication for chemotherapy as initial treatment. Patients could be included regardless of disease stage, age, or Eastern Cooperative Oncology Group status. All patients provided oral informed consent. The study was approved by a local ethics committee.

A structured questionnaire was used to collect clinical, sociodemographic, and QoL data, and was administered orally by paramedical staff to all patients. QoL was assessed using the FACT-B questionnaire after validated translation into Wolof following Functional Assessment of Chronic Illness Therapy^5^ recommendations (Data Supplement), which ensured the validity of the Wolof version of the FACT-B questionnaire. The FACT-B Wolof questionnaire’s internal consistency was confirmed via a Cronbach α score of .6 for the total Wolof FACT-B scale, although several subscales, such as the FACT-B BC subscale (Cronbach α = .37) or emotional well-being subscale (Cronbach α = .52), were less reliable, possibly because of the varied comprehension of items included in these subscales.

The FACT-B questionnaire included 37 questions divided into five domains: Physical Well-Being (PWB; seven questions), Social/Familial Well-Being (SWB; seven questions), Emotional Well-Being (EWB; six questions), Functional Well-Being (FWB; seven questions), and BC Subscale (BCS; 10 questions). The maximum and minimum FACT-B total scores were 138 and 0, respectively. The Trial Outcome Index (TOI) was the sum of three subscales: PWB, FWB, and BCS. TOI aggregate scores have been used for other cancer types and are sensitive indicators of outcome.^6,7^ FACT-B is generally a self-administered questionnaire, but ours was interviewer administered (paramedical staff only) because of literacy issues. Data were collected at three different times: the initial consultation when chemotherapy was prescribed, then 3 months and 6 months after the start of systemic therapy.

Standard protocol in the Le Dantec center was sequential neoadjuvant chemotherapy with four cycles of anthracycline-based regimens followed by four cycles of taxanes. For a metastatic disease, first-line treatment was anthracycline-based chemotherapy (six cycles) followed by second-line taxanes in cases of disease progression (six cycles). Adverse events (AEs) were evaluated on a grading scale of 0 to 5 using the Common Terminology Criteria for Adverse Events, version 5.0. Patient characteristics were summarized using median and interquartile ranges (IQRs) for continuous variables, and numbers and percentages for categorical variables.

Many patients were lost to follow-up, and exploratory analyses were consequently performed to assess the types of missing data before additional investigation. Little’s Missing Completely at Random (MCAR) test was performed using the BaylorEdPsych package, version 0.5, in R (https://cran.r-project.org/web/packages/BaylorEdPsych/index.html). The following candidate variables were included: age and FACT-B total score at baseline, 3 months, and 6 months. In addition, the association between FACT-B total scores at baseline and missing values of FACT-B total scores at 3 and 6 months were assessed using a Student *t* test.

A linear mixed model was used with QoL score as a dependent variable to assess the changes in QoL over time. First, QoL outcome measurements (FACT-B total scores and subscales) over time were compared using participant identification specified as a random effect and time as a fixed effect. Then, linear mixed models were used with FACT-B total and TOI scores as dependent variables, participant identification specified as a random effect, time as a fixed effect, and AE grade, tumor stage, or habitation as a covariate (fixed effect). For each model, we reported the estimated fixed effects (β value) of the independent variable with the associated 95% CI and *P* value. All variables were considered continuous except habitation. According to Eton et al,^8^ the minimally important differences corresponding to the clinical threshold of significance between two QoL scores are as follows: FACT-B, seven points; TOI, five points; and BCS, two points. For all analyses, *P* < .05 was considered statistically significant. All tests were two sided. Statistical analyses were performed using R, version 3.5.2 (The R Foundation for Statistical Computing, Vienna, Austria) and the lmerTest package, version 3.0-1.

## RESULTS

**FIG 1:**
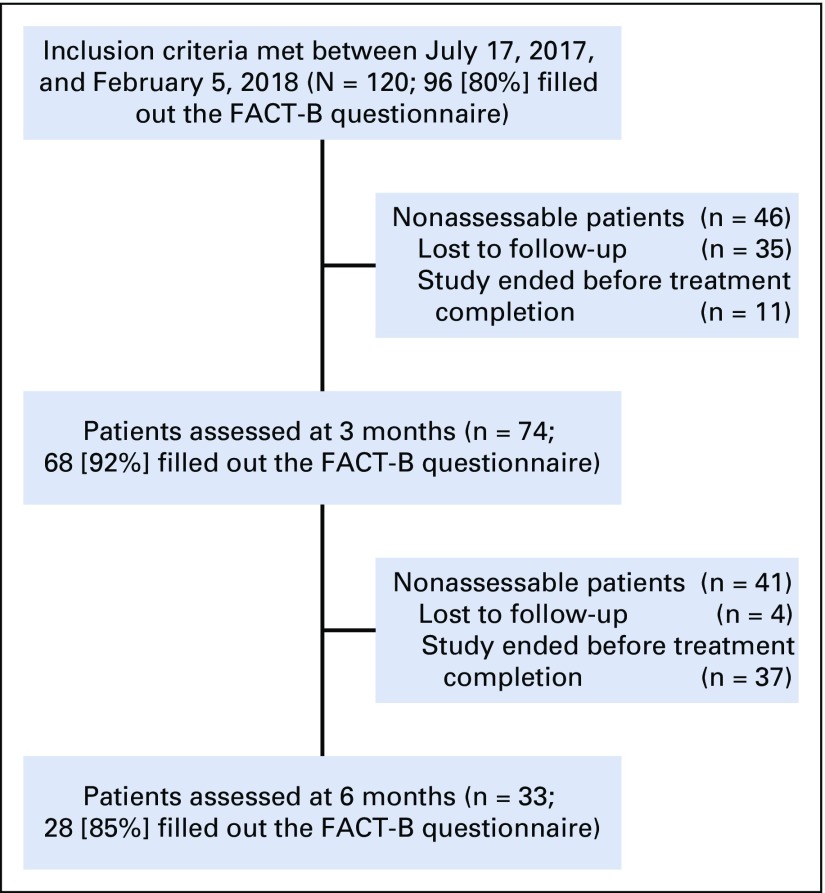
Study flowchart. FACT-B, Functional Assessment of Cancer Therapies-Breast.

A total of 120 patients were included in this study between July 2017 and February 2018 (Fig 1). All patients agreed to participate in the study and to complete follow-up questionnaires.

### Clinical Data

Clinical data are summarized in Table 1. The median age was 45 (IQR, 38-55) years. Most patients (92%) had locally advanced BC (stage T3 to T4), 88% had lymph node involvement (N+), and 50% had a metastatic disease.

**TABLE 1:**
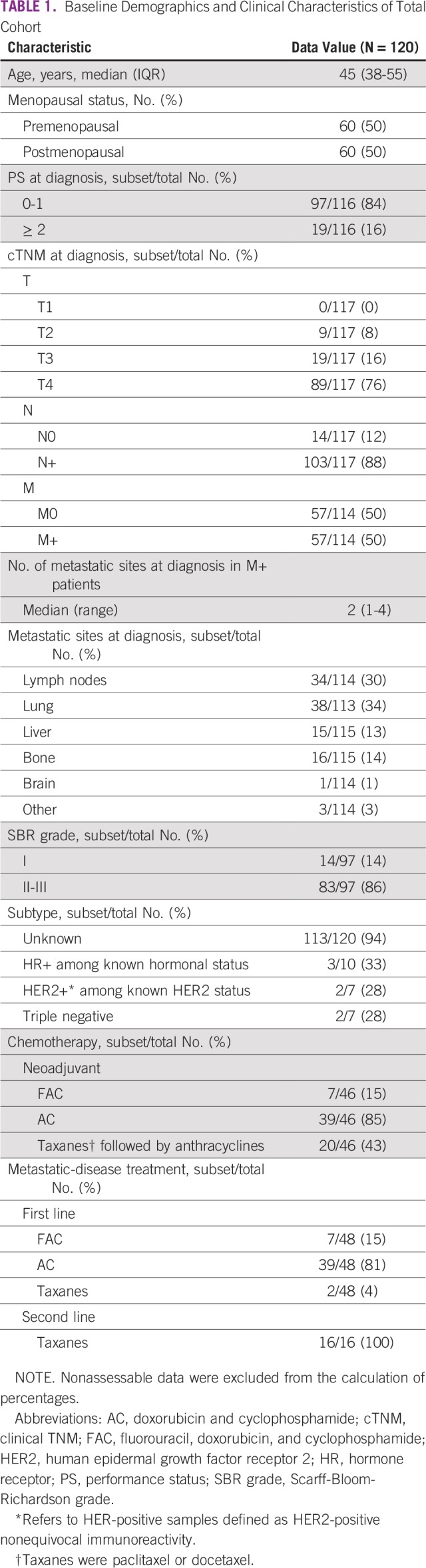
Baseline Demographics and Clinical Characteristics of Total Cohort

In 99% of patients (119 of 120), self-examination was responsible for the initial consultation that led to BC diagnosis. Only one patient was diagnosed via screening mammography. The median time between initial symptoms and the first medical consultation was 10 (IQR, 6-14) months. Thirty-two patients (27%) consulted a traditional healer before conventional medicine. Forty-six patients received neoadjuvant chemotherapy and 48 patients received first-line chemotherapy for metastatic disease; the most frequently prescribed protocol was doxorubicin and cyclophosphamide (AC) in both settings. Twenty-four patients (20%) were lost to follow-up before the first cycle, despite chemotherapy prescription. A median of 54 (IQR, 28.5-96.5) days was observed between the date of diagnosis (biopsy results) and the first day of chemotherapy. Tumor response rates and survival data were unavailable because the follow-up duration was too short and most patients were not assessable after treatment ended. AEs were frequent and of either mild or moderate intensity in most cases (Table 2). The most common AEs that led to discontinuation were nausea, vomiting, and anemia.

**TABLE 2:**
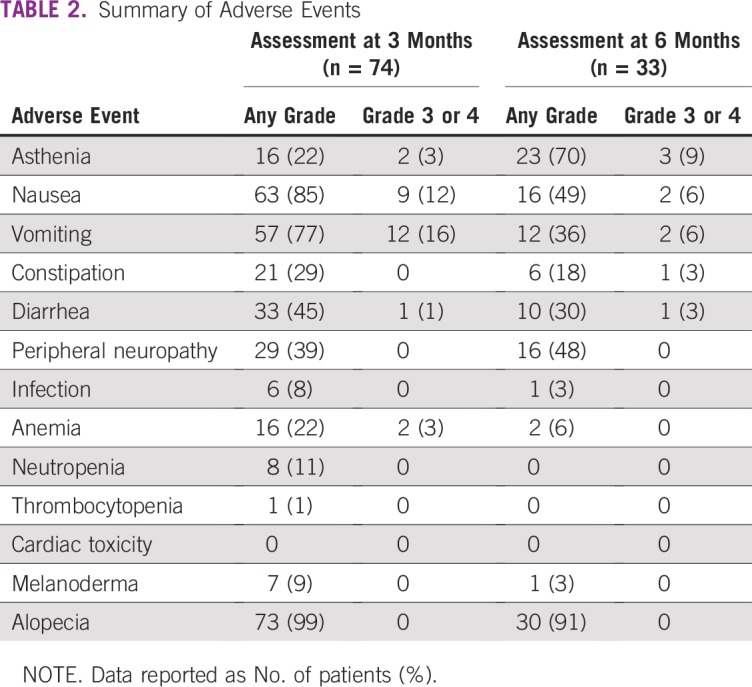
Summary of Adverse Events

### Sociodemographic Data

All sociodemographic data are listed in Table 3. Thirty-three patients (28%) continued working during cancer treatment. Health expenses were mostly paid for by close relatives: 81% of patients received help from family, 15% from friends, and seven patients (6%) had to finance the treatment alone. Only nine patients (7.5%) had medical insurance. At baseline, 36 patients (30%) were already in debt (median, 100,000 CFA francs [XOF; US$171]); after 3 months, 49% of patients were in debt (median, 150,000 XOF [US$257]); and after 6 months, 45% were in debt (median, 160,000 XOF [US$274]).

**TABLE 3:**
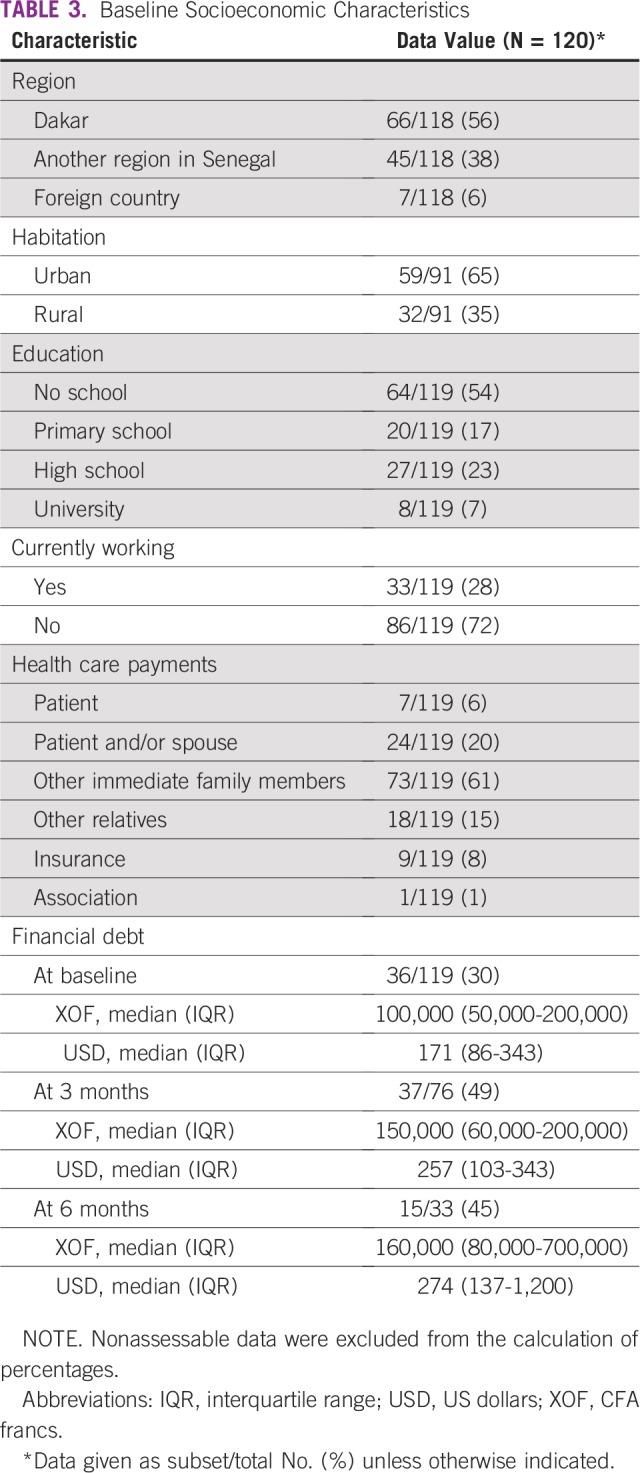
Baseline Socioeconomic Characteristics

### Analyses of Missing Data

The median duration of follow-up was 86 (IQR, 0-138) days. At the 3- and 6-month time points, 46 patients and 41 patients were not assessable, respectively (Fig 1). The mean of FACT-B total scores at baseline was 89.7 for patients who were lost to follow-up at 3 months and 91.5 for patients at 6 months, versus 94.6 for patients with available data at 3 months (*P* = .24) and 97 for patients at 6 months (*P* = .19). The Little’s MCAR test was not statistically significant (*P* = .19) and failed to demonstrate that data were not missing completely at random, which infers that the missing data are independent of patients’ age, TNM stage, or QoL at baseline.

### QoL Data

**FIG 2:**
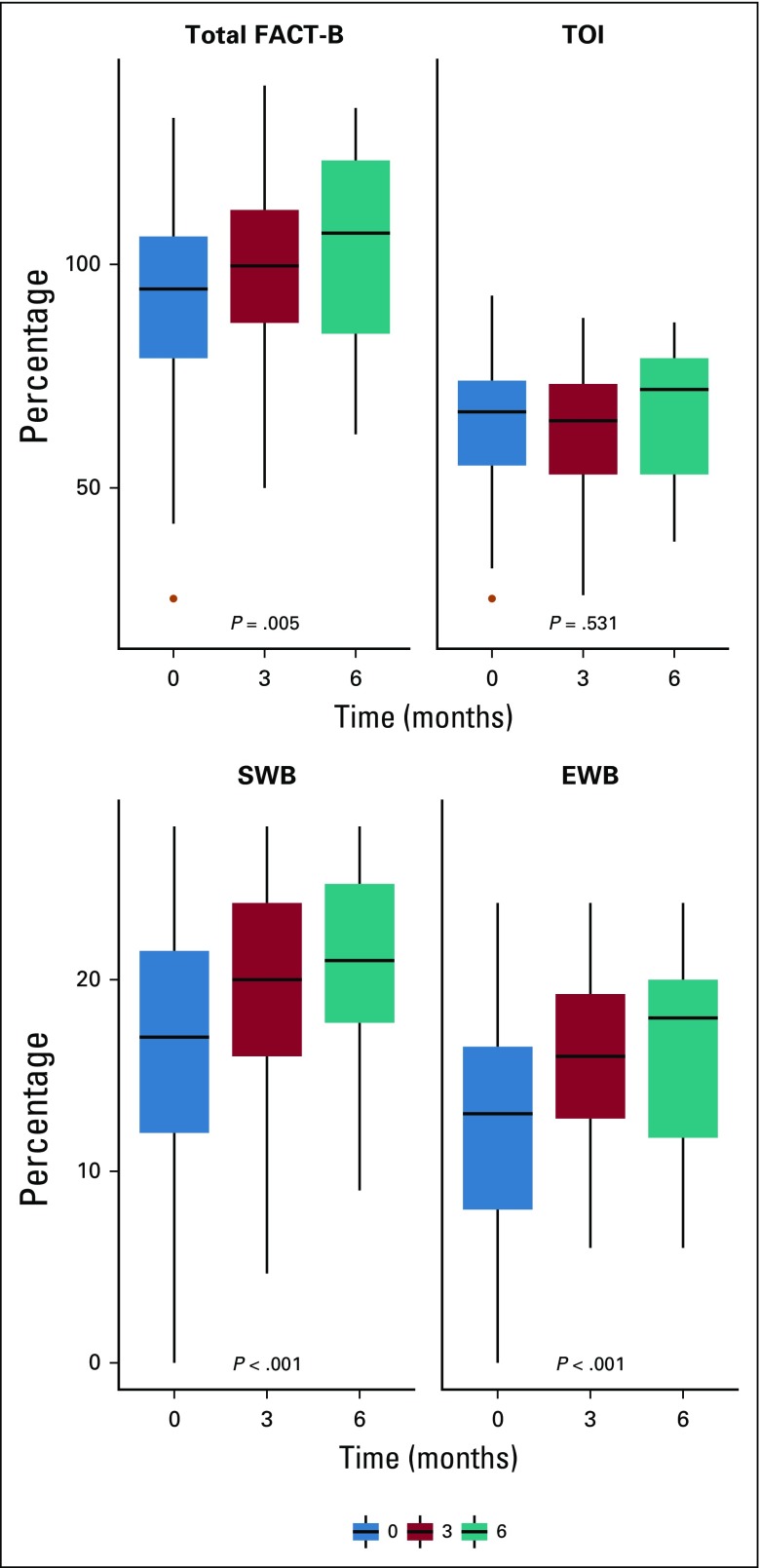
Functional Assessment of Cancer Therapy-Breast (FACT-B) and Trial Outcome Index (TOI) scores over time. Analyses were performed using mixed linear model. FACT-B total is the sum of Physical Well-Being (PWB), Social/Familial Well-Being (SWB), Emotional Well-Being (EMB), Functional Well-Being (FWB), and Breast Cancer Subscale (BCS; maximum score, 138); TOI is the sum of PWB, FWB, and BCS (maximum score, 96). Maximum scores for SWB and EWB were 28 and 24, respectively.

Only patients with BC whose native language is Wolof were assessable for QoL. The numbers of patients that filled out the FACT-B questionnaire were 96, 68, and 28, at baseline, 3 months, and 6 months, respectively (Fig 1). The FACT-B total scores were 94.5 (IQR, 79-106.2), 99.7 (IQR, 86.9-112.2), and 107 (IQR, 84.5-123.2) at the three time points, respectively. FACT-B total scores significantly improved over time (β = 1.58; 95% CI, 0.50 to 2.67; *P* = .005; Fig 2). Likewise, we observed an improvement of SWB (β = 0.91, 95% CI, 0.55 to 1.27; *P* < .001) and EWB scores (β = 0.72; 95% CI, 0.41 to 1.02; *P* < .001; Table 4). However, TOI scores did not significantly change over time (β = −0.23; 95% CI, −0.93 to 0.50; *P* = .53).

**TABLE 4:**
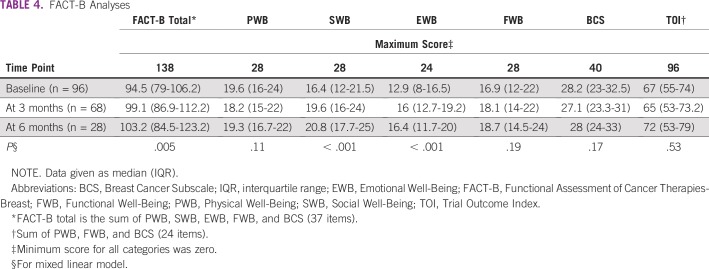
FACT-B Analyses

There was a clinical difference of 8.7 points between the median FACT-B score at baseline and at 6 months. At 6 months, 10 patients (37%) had clinically significant improvements (> 7) in FACT-B total scores compared with baseline. Only five patients (18.5%) had clinically significant improvements (> 5) in the TOI scores at 6 months compared with baseline. Seven patients (26%) and 12 patients (44%) had clinically significant decreases in FACT-B total and TOI scores, respectively, between baseline and 6 months.

**FIG 3:**
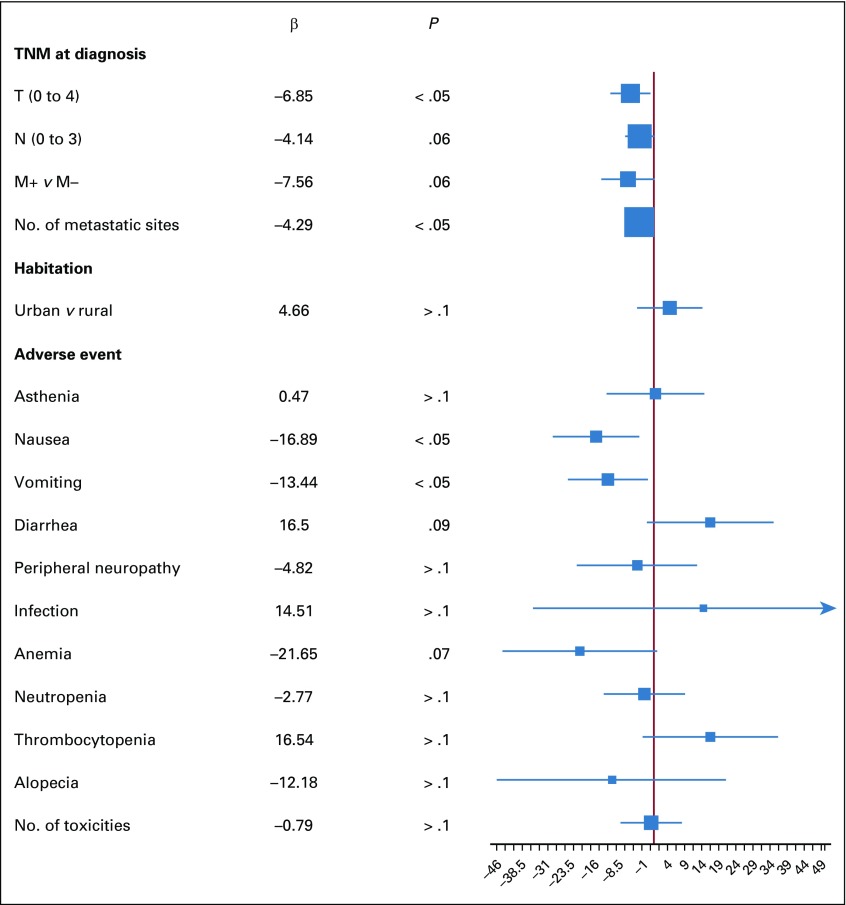
Evolution of total Functional Assessment of Cancer Therapy-Breast score according to tumor stage at diagnosis, habitation, and chemotherapy-related adverse events. Analyses were performed using a mixed linear model.

The association between patient’s QoL and clinical or sociodemographic baseline characteristics was analyzed (Fig 3). First, we found that a higher T stage was significantly associated with poorer FACT-B total and TOI scores (β = −6.85, 95% CI, −12.72 to −0.99, *P* = .02; and β = −5.57, 95% CI, −9.63 to −1.51; *P* = .008, respectively) and a higher N stage was significantly associated only with poorer TOI scores (β = −3.98; 95% CI, −6.98 to −0.97; *P* = .01), whereas the number of metastatic sites was significantly associated with lower FACT-B total and TOI scores (β = −4.29, 95% CI, −7.50 to −1.07, *P* = .01; and β = −3.44, 95% CI, −5.69 to −1.19; *P* < .01, respectively). We then determined that urban or rural habitation did not have a significant association with QoL (*P* = .34 for FACT-B total scores and *P* = .7 for TOI scores). Finally, we evaluated the effects of chemotherapy toxicity on QoL. Two AEs had a significant impact on the FACT-B total scores: nausea (β = −16.89; 95% CI, −29.58 to −4.24; *P* = .012) and vomiting (β = −13.44; 95% CI, −25.15 to −1.72; *P* = .028). The same effects were observed in a mixed model with TOI scores where only nausea (β = −10.89; 95% CI, −19.26 to −2.59; *P* = .013) and vomiting (β = −8.10; 95% CI, −15.83 to −0.34; *P* = .045) were significantly associated with a decrease in TOI scores after 3 and 6 months.

## DISCUSSION

We conducted a prospective, longitudinal study in the Le Dantec University hospital in Dakar, Senegal, to describe the clinical and sociodemographic features of Senegalese patients with BC and to assess their QoL during chemotherapy. Our study population (N = 120 patients) was substantial compared with the entire reported population of women treated for BC in Senegal and seemed to be representative regarding the proportion of spoken language and habitation.

First, patients were significantly younger (median age: 45 years) than North America patients with BC (median age, 62 years, according to SEER registries^9^), but our results are in line with the studies conducted in other African countries.^10-12^ Second, most patients had late-stage disease (50% had stage IV BC) at first visit. Third, QoL longitudinal analysis revealed improvements in FACT-B total scores as medical care continued. However, TOI scores did not improve significantly during the study and more likely reflect clinical treatment tolerance, with more AEs resulting in decreases in TOI scores. The prolonged time between the onset of clinical signs and initial medical visit (median, 10 months), as well as between the date of diagnosis (result of biopsy) and the start of chemotherapy (median, 54 days) should also be emphasized in parallel with the heavy financial burden of cancer treatment (45% of patients were in debt after 6 months).

Age differences at diagnosis between lower- and higher-income countries have been widely discussed in the literature.^13^ Advanced BC stage at diagnosis was a striking feature of the studied population, although it was highly heterogeneous among sub-Saharan African populations.^14^ The late diagnosis of BC is a major issue in low- to middle-income countries.^15^ A median delay of 10 months is in line with results provided in the Espina et al meta-analysis.^16^ As described in bordering countries^17^ and in Senegal,^18^ various factors could contribute to late diagnosis, such as low awareness of BC symptoms, distances to oncology centers from rural areas, insufficient organized screenings, health care costs, and sociocultural factors such as beliefs and traditions that favor initial visits to traditional healers.

The cost of chemotherapy should be considered along with the costs of perfusion sets, supportive care, and the consequences of the loss of a patient’s salary. Currently, no government health insurance program exists for patients with cancer in Senegal, and few patients benefit from private health insurance. The high number of patients lost to follow-up (87 patients after 6 months) may be related to the high cost of cancer treatment and the patients’ and families’ debt loads. To compare, the average salary per month in Senegal is approximately 100,000 XOF (US$170)^19^ and the median debt at 6 months was 160,000 XOF (US$274).

In our study, the TOI scores (67 at baseline) were similar to those of the FACT-B validation study conducted in North America (mean, 66.8),^3^ though the mean FACT-B total scores were lower in our study (112.8 in the validation study; 94.5 at baseline in our cohort). These results should be interpreted with caution regarding a potential bias for length of time and cultural differences in the perception of QoL.

In western populations, QoL improvements during chemotherapy for metastatic BC have already been observed.^20^ However, in the adjuvant or neoadjuvant setting, patients in westernized countries are mostly asymptomatic and chemotherapy has reportedly led to a decrease in QoL.^21^ In our study, QoL improvements may be related to both the effectiveness of chemotherapy in this population with high tumor burden and the strong psychological familial support. Indeed, EWB and SWB are two dimensions that improved significantly over the time, which is supported by the proportion of patients whose FACT-B total scores increased after 6 months (37%); only half had a meaningful improvement of TOI scores. On the other hand, advanced-stage cancer at baseline was significantly associated with a decreased QoL (FACT-B and TOI scores), and every patient with a meaningful decrease of FACT-B total score (26%) at 6 months compared with baseline had a meaningful decrease in TOI score, and a decreased TOI score was significantly associated with nausea and vomiting, which could lead to treatment discontinuation. This observation suggests that the degradation of QoL may be mainly explained by physical decline (eg, poor tolerance to chemotherapy, progressive disease). Indeed, access to antiemetics is limited in Senegal. Optimizing nausea and vomiting prevention (ie, systematic prescription of antiemetics, using corticosteroid therapy, improving accessibility to serotonin blockers, permitting the use of neurokinin-1 inhibitors in the country), should significantly improve chemotherapy tolerance and QoL of patients with BC in Senegal.

In this study, we prospectively assessed the QoL of Senegalese patients with BC. Translating the FACT-B questionnaire was a key preliminary step and its psychometric properties were verified before publishing the study. Of note, the FACT-B questionnaire has already been translated into other African languages,^22^ but in this study, we have provided a longitudinal assessment of QoL in an African population during anticancer systemic treatment.

One major limitation of our study was the lack of tumor-response rates and survival data, due to a short follow-up duration and numerous patients lost to follow-up; the latter was mainly attributable to high treatment cost. Moreover, we cannot exclude a selection bias, because our study was monocentric, and includable patients may have been missed because of logistical issues. Another potential selection bias was that the FACT-B questionnaire was administered by an interviewer instead of the patients themselves, because of literacy issues. However, the questionnaire was administered by nurses previously trained for QoL interviewing, and not the physicians in charge, which may partially limit this bias.

Both the relatively small number of enrolled patients and the high rate of patients lost to follow-up led to a high attrition rate. Forty-eight patients were lost due to the closure of the study before treatment completion. Importantly, there was no significant difference in FACT-B scores at baseline between patients with and without follow-up, and Little’s MCAR test failed to point out missing data as not MCAR. Taken together, these data suggest that missing values were not associated with QoL at baseline. In this setting, the use of a linear mixed model for analyses was particularly appropriate because it accounts for missing values quite well.^23^ Therefore, the major limitation related to the high number of missing values was a low-power analysis. The high number of nonassessable patients highlights the difficulty of managing oncological treatments in low- to middle-income countries.

Although QoL scales cannot embrace the many factors that may influence actual QoL, our study provides deeper insight into demographics and QoL data of patients with BC in Senegal. We demonstrate improvements in QoL even in cases of advanced disease treated by suboptimal chemotherapy protocols and limited supportive care. Organized BC screenings,^24^ early BC treatment, and supportive care optimization could decrease medical costs and improve QoL.
